# Changes of Signaling Pathways in Hypothalamic Neurons with Aging

**DOI:** 10.3390/cimb45100523

**Published:** 2023-10-12

**Authors:** Petr M. Masliukov

**Affiliations:** Department Normal Physiology, Yaroslavl State Medical University, ul. Revoliucionnaya 5, 150000 Yaroslavl, Russia; mpm@ysmu.ru

**Keywords:** hypothalamus, aging, signaling pathways

## Abstract

The hypothalamus is an important regulator of autonomic and endocrine functions also involved in aging regulation. The aging process in the hypothalamus is accompanied by disturbed intracellular signaling including insulin/insulin-like growth factor-1 (IGF-1)/growth hormone (GH), phosphatidylinositol-4,5-bisphosphate 3-kinase (PI3K)/protein kinase B (AKT)/the mammalian target of rapamycin (mTOR), mitogen activated protein kinase (MAPK), janus kinase (JAK)/signal transducer and activator of transcription (STAT), AMP-activated protein kinase (AMPK), nuclear factor kappa-light-chain-enhancer of activated B cells (NF-ĸB), and nitric oxide (NO). In the current review, I have summarized the current understanding of the changes in the above-mentioned pathways in aging with a focus on hypothalamic alterations.

## 1. Introduction

The hypothalamus is the regulatory hub controlling homeostasis, reproduction, circadian rhythms, and the endocrine system [1,2]. At the end of the XXth Century, it was proposed that hypothalamus participates in the aging regulation. According to this theory, age-related raising of the hypothalamus threshold for homeostatic signals leads to aging and the appearance of age-related disease in different species [3,4]. The advancement of new research techniques, including cutting-edge approaches in genetics, molecular biology, and neuroscience, allowed the study of the role of the hypothalamus in aging mechanisms in more detail [5,6,7]. In recent years, it has been suggested that the age-related loss of hypothalamic stem cells plays a major role in the development of hypothalamic neuroinflammation and mammalian aging [8,9].

The process of aging is associated with many chronic pathological conditions such as vascular diseases, diabetes mellitus, cancer, and metabolic syndrome. It has been established that aging is accompanied by disturbances of signaling cascades including insulin/insulin-like growth factor-1 (IGF-1)/growth hormone (GH), phosphatidylinositol-4,5-bisphosphate 3-kinase (PI3K)/protein kinase B (AKT)/the mammalian target of rapamycin (mTOR), mitogen activated protein kinase (MAPK), janus kinase (JAK)/signal transducer and activator of transcription (STAT), AMP-activated protein kinase (AMPK), and nuclear factor kappa-light-chain-enhancer of activated B cells (NF-ĸB). These pathways regulate energy balance, cellular plasticity, mechanisms supporting homeostasis, growth, reproduction, and inflammation [10,11,12]. However, many studies of intracellular signaling pathways in aging focus on changes in peripheral organs, while little attention is paid to hypothalamic alterations.

## 2. A Brief Description of the Main Hypothalamic Nuclei with a Focus on Mediobasal Hypothalamus

With rare exceptions (supraoptic (SON) and paraventricular (PVN) nuclei), the majority of hypothalamic nuclei have obscure boundaries. They are no longer thought of as centers with strictly limited functions. PVN and SON nuclei control different physiological functions including stress and the adaptive responses of the organism. The PVN has a non-uniform cellular composition because it contains magnocellular, parvocellular, and neurons with distally projecting axons. The PVN regulates stress reactions, metabolism, growth, reproduction, and activity of the autonomic nervous system. Neurons of the magnocellular SON regulate salt and water homeostasis together with blood pressure [12,13].

The lateral hypothalamic region (LHA) and other hypothalamic nuclei, including the arcuate (ARN), ventromedial (VMN), and dorsomedial (DMN) nuclei, are all involved in regulation of metabolism. In addition, the ARN controls growth and sex gland function together with the median preoptic (MPN) and anteroventral periventricular (AVPV) nuclei [14,15]. The suprachiasmatic nucleus (SCN) is the primary pacemaker of circadian rhythms [16]. The tuberomamillary nucleus is known as the important wake-promoting center and participates in the regulation of temperature, learning, sleep, and behavior [17].

**Figure 1:**
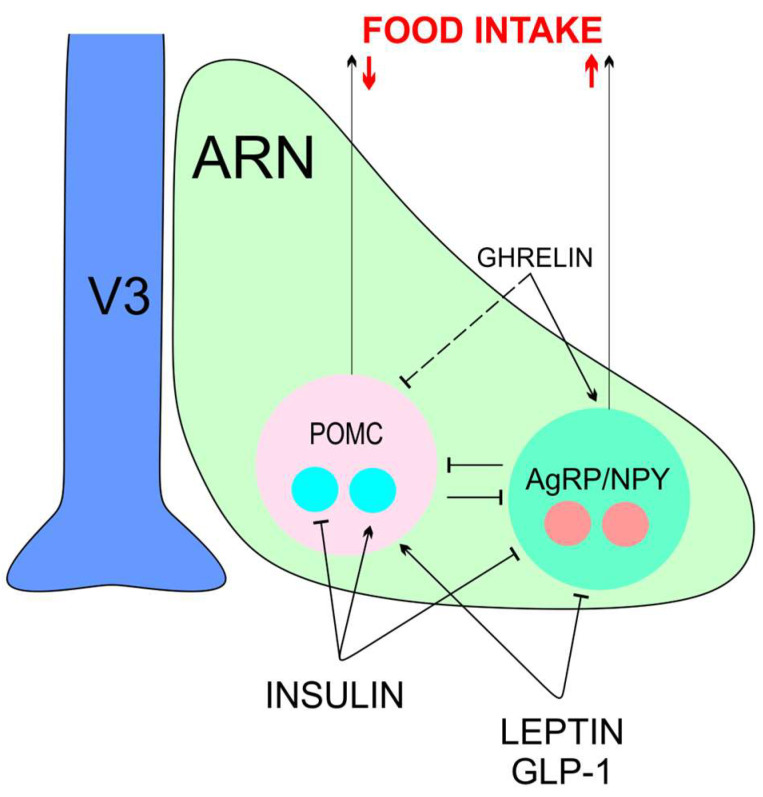
The scheme of orexigenic (AGRP/NPY) and anorexigenic (POMC/CART) ARN neurons and their regulation by circulating hormones. ARN—the arcuate nucleus, AGRP—agouti-related peptide, GLP-1—glucagon-like peptide-1, NPY—neuropeptide Y, POMC—proopiomelanocortin. Red arrows indicate decrease or increase in food intake.

The VMN and ARN are combined into the mediobasal hypothalamus based on their location and functions, such as their role in regulating feeding and sexual behavior [18,19]. From data of D. Cai’s group, this region is extremely important for the control of whole-body aging [5]. In the ARN, proopiomelanocortin (POMC)/cocaine-amphetamine-regulated transcript (CART) neurons and agouti-related peptide (AGRP) neuropeptide Y (NPY) neurons counteractively regulate feeding. POMC and CART have anorexigenic action and increase energy expenditure. NPY and AGRP stimulate feeding behavior (Figure 1). Insulin, together with the leptin, stimulates POMC/CART neurons and inhibits NPY/AGRP ones. Conversely, ghrelin stimulates NPY/AGRP neurons and inhibits POMC/CART cells [15,20]. In contrast to leptin, insulin inhibits the activity of some POMC neurons. 

**Figure 2:**
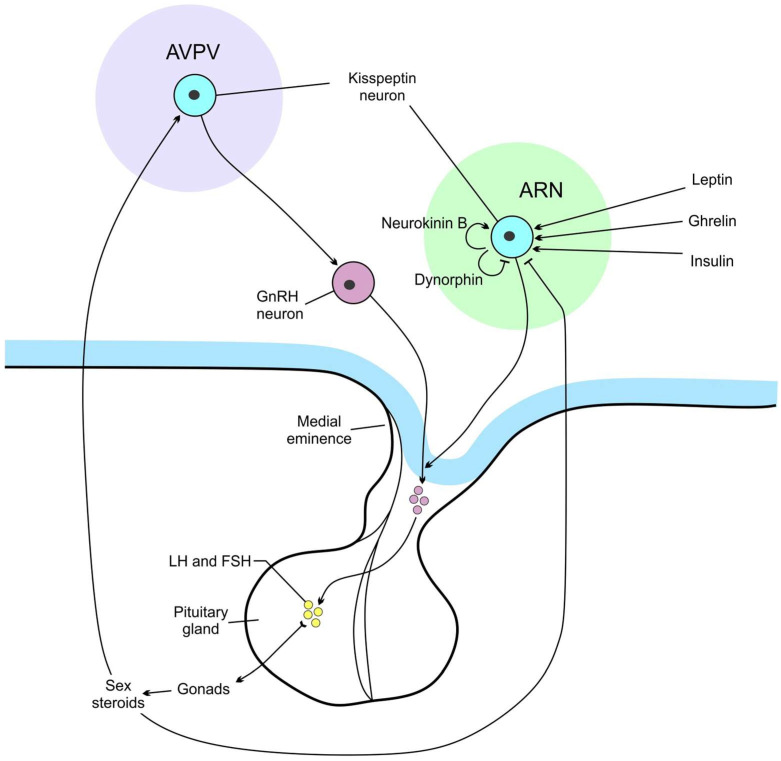
The scheme of two main populations of kisspeptin neurons. ARN—the arcuate nucleus, AVPV—anteroventral periventricular nucleus, FSH—follicle stimulating hormone, LH—luteinising hormone.

Neurons secreting growth hormone-releasing hormone (GHRH) are found in the ARN [21], and those expressing GH-inhibitory hormone (somatostatin, SS) are located in the anterior periventricular, ARN, VMN, LHA, PVN [22]. The control of reproduction depending on the level of nutrients is mediated by kisspeptin-secreting neurons which regulate gonadotropin releasing hormone (GnRH)-producing neurons (Figure 2). Two populations of kisspeptin synthesizing neurons are located in the hypothalamus, in the ARN, and AVPV nuclei [23]. These ARN neurons, known as KNDy neurons, contain two stimulatory peptides, kisspeptin and neurokinin B (NKB), and an inhibitory peptide, dynorphin [24]. KNDy neurons mediate negative steroid feedback to the GnRH neurons and generate GnRH episodic release, whereas AVPV kisspeptin neurons act as positive feedback mediators and are responsible for GnRH/LH surges [23]. Mammalian GnRH neurons form a dispersed population throughout the rostral forebrain. Most GnRH neurons are observed in the medial septal region, the rostral preoptic area, and the anterior hypothalamic area [25].

In addition to leptin, ghrelin, and insulin, incretin hormones including glucagon-like peptide-1 (GLP-1) and glucose-dependent insulinotropic polypeptide (GIP) also control appetite and body weight (Figure 1 and Figure 2), and act on their cognate receptors GLP-1R and GIPR [26]. GLP-1Rs are expressed throughout the brain, majorly in the solitary tract nucleus, ARN, PVN, and DMN of the hypothalamus, along with central amygdale [27]. Intracerebroventricular injection of GLP-1 inhibits eating and reduces body weight. GLP-1R agonists can inhibit eating through activation of vagal afferent GLP-1Rs and via a direct effect on the brain [28].

## 3. Insulin/IGF-1/GH Signaling

The insulin-like growth factor (IGF) family includes three known ligands (IGF-1, IGF-2, and insulin). In contrast to invertebrates, mammals, such as mice and humans, have two distinct receptors for insulin and IGF-1, each of which is encoded by a separate gene. While insulin primarily controls the metabolism, IGFs are responsible for long-lasting effects on the regulation of growth, development, and differentiation of cells and tissues [29].

Insulin is synthetized by beta cells in the pancreas and crosses the blood–brain barrier by a saturable mechanism. Insulin in the CNS has opposite effects compared to the periphery, increasing blood glucose levels, decreasing feeding and body weight, and even decreasing the blood levels of insulin [30]. Some recent data indicate that brain insulin downregulates expression of genes involved in glucose metabolism, reduces oxidative stress, regulates the expression of glutamate receptors, upregulates GABA receptors, and suppresses multiple neuropeptides, which contribute to synaptic plasticity and neuronal activity, particularly in the hypothalamus [31].

In vitro biological effects of IGFs are relatively weak and often are observed in the presence of other hormones or growth factors. Possibly, IGFs act as permissive factors to augment the signals of other factors [32].

Insulin can bind to the IGF-1 receptor (IGF-1R) but IGFs bind less well to the insulin receptor (IR) [32]. When IGF-1Rs and IRs are produced in the same cells, some of them form a hybrid receptor [33]. Although structurally, the IGF-1R and the IR are highly homologous, the homology of their C-terminal regions is relatively low [32,33]. IGF-1Rs and IRs are found throughout the CNS including the hypothalamus, and their relative distributions vary, but overlap [34,35]. IRs’ expression is higher than that for IGF-1Rs in the ARN [30]. However, there are no data about changes in the number of IGF-1Rs and IRs in different hypothalamic nuclei with aging. 

In contrast to the invertebrate system, mammalian IGF-1 production is regulated by GH secretion from the pituitary. In vivo, many physiological effects of GH are mediated by GH-induced hepatic IGF-1 and local IGF-1 [36]. The secretion of GH is stimulated by GHRH and ghrelin and inhibited by SS. GH exerts its effects by interacting with the GH receptor (GHR), a member of the cytokine receptor superfamily. The GHR is expressed in multiple tissues, including muscle, adipose, heart, kidney, intestine, and bone, with expression being most abundant in the liver [37]. GH secretion is inhibited by IGF-1 in a feedback loop and modulated by other hormones including insulin [38]. Short-term treatment with GH results in rapid insulin-like effects, including the stimulation of lipogenesis and inhibition of lipolysis [39]. However, long-term use of GH counteracts the effects of insulin and induces insulin resistance and diabetes [40,41].

**Figure 3:**
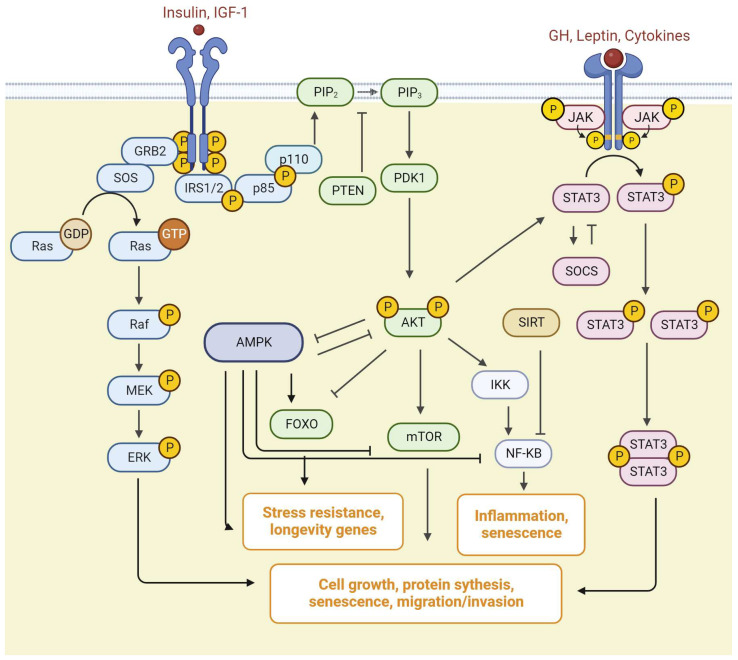
The basic scheme of insulin/IGF-1/GH, PI3K)/AKT)/mTOR, MAPK, JAK/STAT, AMPK, and NF-ĸB pathways. AKT—protein kinase B, AMPK—AMP-activated protein kinase, ERK—extracellular signal-regulated kinase, FOXO—forkhead box O, GH—growth hormone, GRB2—growth factor receptor-bound 2, IGF-1 -insulin-like growth factor-1, IKK—inhibitor of nuclear factor kappa B kinase, IRS—insulin receptor substrate, JAK—janus kinase, MEK—mitogen activated protein kinase/ERK kinase, mTOR—the mammalian target of rapamycin, NF-ĸB—nuclear factor kappa-light-chain-enhancer of activated B cells, PDK—phosphoinositol-dependent protein kinase, p85 and p110—subunits of the phosphatidylinositol-4,5-bisphosphate 3-kinase, PIP_2_—phosphatidylinositol 4,5-bisphosphate, PIP_3_—phosphatidylinositol-3,4,5-triphosphate, PTEN—phosphatase and tensin homolog deleted on chromosome 10, SIRT—sirtuins, SOCS—suppressor of cytokine signaling, STAT—signal transducer and activator of transcription.

IR signaling is mediated by the action of insulin receptor substrate (IRS) on two canonical pathways, the phosphatidylinositol-4,5-bisphosphate 3-kinase (PI3K)/protein kinase B (AKT)/the mammalian target of rapamycin (mTOR) signaling pathway, and Ras/mitogen activated kinase (MAPK) cascades (Figure 3) [42]. The PI3K/AKT/mTOR signaling pathway is critically important in the regulation of different biological processes such as cell cycle, metabolism, and signal transduction [43,44,45]. 

GH binding with GHR activates the tyrosine kinase janus kinase 2 (JAK2), which phosphorylates several tyrosine residues on the intracellular domain of the receptor and initiates a multitude of signaling cascades (Figure 3). Activation of the GHR/JAK2 complex leads to activation of signal transducers and activators of transcription (STATs), ERK/MAPK, and PI3K/AKT pathways [37,46].

GH and IGF-1 levels in the blood peak in adolescence and then progressively decline, becoming scarcely detectable in the elderly [47]. The level of GHRH in the median eminence decreases in aged rats, while the SS level in the median eminence, as well as GHRH and SS level in the neuronal somata remains intact [48]. It was proposed that SS-induced inhibition of GH was sensitized in aged rats, indicating a possible cause for the age-related decrease in GH level in plasma [49]. However, the percentage of SS-immunoreactive neurons in the mediobasal hypothalamus, as well as their number, does not change in males and females with aging [22]. 

In both humans and mice, a substantial increase in GH levels is associated with high risks of diseases and reduced life expectancy [50]. To date, the mechanism of age-related decline in GH and IGF-1 levels remains unclear [51].

Reduced activity of the insulin/IGF1 signaling increases longevity, improves health in aged animals from worms and flies to mice [52,53,54]. However, global reduction in insulin signaling by the deletion of IR, IGF1R, or IGF1 leads to early mortality [55,56]. Total disruption of insulin signaling also has defects such as metabolic syndrome, and reduced body size and fertility [57]. Total deletion of IR receptors in peripheral tissues after adulthood leads to a significant reduction in male life span [58]. On the other hand, insulin in the brain acts as a neuroprotector, lowering damage induced by ischemia, β-amyloid toxicity, oxidative stress, and apoptosis [59]. Brain insulin resistance and low levels of brain insulin often lead to metabolic and cognitive dysfunctions, including obesity, type 2 diabetes, and Alzheimer’s disease [60,61]. 

Reduced expression of the IGF-1R in the C57BL/6 mice caused only a 5% prolongation of lifespan in females [62]. However, its partial reduction in fat can lead to lifespan extension [63]. 

Hypothalamic insulin resistance caused by overnutrition occurs more rapidly than in other insulin-sensitive tissues [64]. Nevertheless, selective ablation of IR in the different populations of hypothalamic neurons, including AGRP and POMC cells, has only a moderate effect on energy balance [65,66]. In addition, mice lacking the IR in the MCH neurons of the LHA had a lean phenotype and exhibited improved locomotor activity and insulin sensitivity under a high-fat diet [67]. 

There is a link between the IGF-1 and GLP-1 signaling pathways. GLP-1, apart from regulating food intake, prevents neuronal death mediated by amyloidogenesis, cerebral glucose deprivation, neuroinflammation, and apoptosis through modulation of PI3/Akt mTOR and MAPK/ERK [68]. GLP-1 downregulation causes oligodendrocyte deterioration, demyelination, glial hyperactivity, immunological dysregulation, and neuroexcitation in the brain [69]. IGF-1 resistance and GLP-1 deficiency impair protective cellular signaling mechanisms, contributing to the progression of neurodegenerative diseases [68,70]. The anti-inflammatory effect of GLP-1 was lost in astrocytes with IGF1-R knockout [71].

However, there are only few data about changes in GLP-1 signaling in aging. The anorexic effect of GLP-1 was attenuated in aged mice [72], thus highlighting the alterations of hypothalamic sensitivity to hormones and environmental factors with aging. 

## 4. PI3K/mTOR/AKT Pathway

The binding of IGF-I or insulin to the α subunit of its specific receptor on a target cell membrane leads to the conformational change in the β subunit, resulting in the activation of receptor tyrosine kinase activity [31,73]. The activated receptor in turn phosphorylates specific substrates, in particular insulin receptor substrate (IRS) and Src homology collagen (Shc) [32]. It is known that mice have four IRS proteins (IRS1-IRS4) but IRS3 is absent in humans [74]. Tyrosine phosphorylated substrates (pIRS-1, pIRS-2, pShc, pGab1) are recognized by some SH-2 domain containing molecules, including growth factor receptor-bound 2 (Grb2), a p85 regulatory subunit of PI3-kinase, and SHP2/Syp. Grb2 is required for IGF-I-induced Ras-MAPK pathway activation (Figure 3) [32]. The interaction between IRS and SHP-2 is important for the crosstalk between IGF-I and the integrin pathway [75]. 

The PI3K family includes three classes, from I to III [76]. PI3K itself is a heterodimer consisting of regulatory p85 and catalytic p110 subunits. At rest, it is in an inactive state in the cytoplasm of the cell. The p85 regulatory domain of PI3K (p85-PI3K) binds the phosphorylated domains of IRS-1, activating PI3K [77]. The activated enzyme catalyzes the synthesis of phosphatidylinositol-3,4,5-triphosphate (PIP3) from the membrane phosphatidylinositol 4,5-bisphosphate (PIP2). PIP3 provides anchoring of phosphoinositol-dependent protein kinase (PDK-1/2) in the membrane. Membrane-bound PDK-1/2 is activated and, in turn, catalyzes the phosphorylation of inactive AKT. The activation of PI3K induces AKT phosphorylation at two residues: Thr 308 in the kinase domain and Ser473 in the hydrophobic motif. Phosphorylated AKT (pAKT) acquires catalytic properties, dissociates from the membrane, and provides phosphorylation of various intracellular proteins in the cytoplasm and nucleus [78,79]. AKT regulates the activity of some longevity genes, such as mTOR, NF-κB and forkhead box O (FOXO). AKT stimulates mTOR and NF-κB, and inhibits FOXO factors (Figure 3) [80].

An important downstream target of AKT is mTOR. mTOR is a serine/threonine protein kinase that is a part of two different protein complexes: the mTOR complex 1 (mTORC1) and the mTOR complex 2 (mTORC2). mTORC1 is inhibited by rapamycin, promotes protein synthesis and autophagy, and integrates hormonal and environmental signals. mTORC2 is involved in the insulin signaling pathway. In addition to mTOR protein kinase, mTORC1 includes RAPTOR protein and AKT PRAS40 substrate; mTORC2—RICTOR protein and other specific protein subunits such as mSin1 and Protor-1/2. In addition, both mTORC1 and mTORC2 also have the same components: mLST8/GβL as well as the DEPTOR regulatory protein [81].

With aging, the PI3K/AKT/mTOR signaling pathway dysregulates in many tissues including the brain and hypothalamus. There are some reports about changes in separate parts of this system, and selective action may reduce aging manifestations. 

IRS-1 and IRS-2 are functionally different. IRS-1 knockdown or overexpression did not affect the IGF signals but IRS-2 knockdown impaired the IGF-1 signals and IGF bioactivities [82,83]. Age-related diseases such as obesity, skin pathology, osteoporosis, sarcopenia, and glucose intolerance were less common in IRS-1 complete knockout mice [84]. Nevertheless, the lifespan of mice was not increased by selective IRS-1 deletion in the liver, muscles, fat, or neurons. Compared to IRS1 deletion in the muscle, liver, and fat, neuron-selective IRS1 knockout increased energy consumption, movement activity, and insulin responsiveness, particularly in aged male mice [85].

There are contradictory data on the role of IRS-2 in aging regulation. Taguchi et al. (2007) reported that mice heterozygous for a null mutation in the IRS-2 in the whole body or separately in the brain had an increased life span [86]. However, using the same mouse model, Selman et al. (2008) observed no evidence for life-span extension and even shortened survival time [87]. It is suggested that IRS-1 is more involved in mitogenic signaling whereas IRS-2 is more important in the regulation of metabolism [88]. In this case, a more severe lifespan-prolonging effect in IRS-1 mutants could be due to lowered cell division while IRS-2 mutants may not demonstrate lifespan extension due to metabolic disturbances [58].

Leptin and insulin action can differently modulate PI3K activity in hypothalamic neurons. It is important for metabolic syndrome, which is a common manifestation of aging [89,90,91]. In the ARN, leptin directly activates PI3K in POMC neurons, but indirectly inhibits it in AGRP neurons [92]. Insulin, vice versa, stimulates PI3K signaling in both POMC and AGRP neurons [93]. Selective inactivation of PI3K reduced leptin-stimulated excitation of hypothalamic POMC neurons in the ARN and caused suppression of food intake [94]. Leptin and insulin influence to decrease food intake was interrupted by PI3K inhibitors [95]. In turn, pharmacological inhibition of PI3K eliminated insulin-induced activation of mTOR [96]. However, the exact role of PI3K in hypothalamic neurons in the regulation of metabolism during aging has yet not been fully established. In C. elegans, mutants lacking any active PI3K are extremely long lived and stress resistant [97]. 

Despite many studies having demonstrated that aging is closely related to the PI3K/AKT signaling pathway, the detailed mechanism underlying it is still not completely understood. Often constitutive activation of AKT signaling leads to tissue overgrowth and is frequently observed in cancer cells, whereas reduced AKT activity is associated with diabetes and growth defects [98,99]. In aging, the occurrence of diabetes and obesity is associated with insulin resistance [100], which leads to the downregulation of AKT and upregulation of FOXOs. In addition, AKT demonstrates differential actions in separate organs [101,102].

There are different results concerning AKT modulation and aging in the brain. Some studies showed a reduction in AKT activity, others found an increase in its phosphorylation status with the aging process [103,104]. In addition, there are contradictory data on AKT expression in Alzheimer’s disease with evidence of increased and decreased AKT activity [105,106]. In the hypothalamus of rodents, the expression of AKT does not change or there are small disturbances with aging [107,108]. On the other hand, activation of the PI3K/AKT/mTOR pathway in the hypothalamus leads to a comparable weight increase, and obesity is often observed with aging [92].

AKT phosphorylates FOXO, induces its nuclear translocation, and inhibits its action in vitro and in vivo. FOXO performs a considerable role in aging and age-related diseases including neurodegenerative and oncological pathology [109]. FOXO overexpression promotes longevity in many species including mammals by combined autocrine and paracrine effects [108]. However, there are contradictory facts that FOXO activity elevates with aging. Tumor necrosis factor (TNF)-α raises the FOXO1 activity by preventing its phosphorylation. Enlarged FOXO1 activity suppresses the *gnrh1* gene and activates the NF-κB inflammatory signaling, preventing GnRH secretion [110]. 

Hypothalamic FOXO1 increases food intake and body weight by stimulating the transcription of orexigenic NPY/AGRP and suppressing POMC neurons in the ARN [45,111,112]. Nevertheless, dietary disturbances are not necessarily associated with the changes in PI3K/AKT/mTOR and FOXO signaling [112].

Different studies have shown that inactivation of mTOR signaling can prolong the duration of life, which might explain the lifespan extension of mice with rapamycin therapy [113,114]. Diet-induced hyperactivation of mTORC1 induces metabolic disturbances, including obesity and type 2 diabetes, as well as cancer and neurodegenerative diseases [115,116,117]. Upregulation of mTORC1 and mTORC2 inhibitory protein DEPTOR in hypothalamic neurons reduced obesity and improves glucose metabolism [118]. Nevertheless, the expression of mTOR in separate hypothalamic nuclei is different through aging. The proportion of mTOR-IR decreased in DMN and VMN neurons and increased in the ARN in 12-month-old and 24-month-old rats [119].

Loss of RICTOR, a part of the mTORC2 complex, results in a sex-specific reduction in male life expectancy even when the intervention is initiated at maturity, in contrast to deletion of IRS1 [120]. Moreover, both male and female mice exhibited a decreased lifespan, activity, glucose tolerance, and insulin responsiveness after knockout of RICTOR in the hypothalamus [121].

In addition to mTOR, AKT through different pathways could trigger the activation of the NF-κB system, which inhibits apoptosis, autophagy, and stimulates inflammatory responses, which is very important for aging [122]. 

## 5. Ras/Raf/MEK/ERK Pathway

The mitogen-activated protein kinases/extracellular signal-regulated kinase (MAPK/ERK) pathway is reported to be associated with cell proliferation, differentiation, migration, senescence, and apoptosis. Insulin is also able to modulate cell growth and proliferation through induction of Shc and its downstream targets Ras, ERK, and mitogen-activated protein kinase (MAPK). In the Ras/MAPK signaling pathway, activated IRSs interact with growth factor receptor-bound protein 2 (Grb2). The tyrosine 891 in IRS-1 is known to be a Grb2 binding site phosphorylated by IGF/insulin stimuli, which is important for the activation of the Ras-MAPK cascade [123]. Grb2, in turn, can activate MAPK cascades via an interaction with the Ras guanine nucleotide exchange factor SOS. Subsequently, SOS activates the small G protein Ras, which in turn recruits MAPK3, also known as Raf kinase. The Raf kinase phosphorylates and activates a MAPK/ERK kinase (MEK1 or MEK2). The MEK phosphorylates and activates a mitogen-activated protein kinase (MAPK). MAPK is referred to as extracellular signal-regulated kinase (ERK). ERK (MAPK) further phosphorylates different proteins in the cytoplasm or transcription factors in the nucleus (Figure 3) [124]. 

This pathway is more often activated by IGF-1R compared to IR [125]. Moreover, different IRα isoforms stimulate specific downstream pathways: the IR-A isoform better activates the Shc/Ras/ERK/MAPK signaling, while the IR-B stimulates PI3K/Akt/mTOR [126,127].

There is evidence of ERK/MAPK impairment in the brain pathology. ERK/MAPK stimulation decreases neuronal injury following ischemia-hypoxia [128]. Treatment of brain-derived neurotrophic factor (BDNF) leads to elevation of ERK/MAPK activity during 12 h after ischemic-hypoxic injury. Resveratrol and fisetin also activate ERK/MAPK and inhibit cell death in a model of Huntington’s disease [129]. 

During aging, the expression of phosphorylated ERK1/2 and MAPK was reduced in the rat cerebral cortex, striatum, and hippocampus [130]. However, ERK1/2 phosphorylation was increased in the hypothalamus of aged rats [131].

In the hypothalamus, insulin decreases mRNA expression of orexigenic neuropeptides NPY and AgRP through an ERK/MAPK dependent mechanism, [132]. Obese mice also exhibit an increased hypothalamic expression of the MAPK phosphatase 3 associated with a reduced phosphorylation of ERK [133,134]. 

In the SCN of the hypothalamus, nocturnal light activates ERK/MAPK [135], and inhibition of ERK/MAPK prevents the light response [136]. Thus, ERK/MAPK is an important signal pathway involved in clock function. Rhythmicity in the SCN declines with aging, and impairment in the ERK/MAPK pathway can play an important role in this process. However, there are no direct data about changes in the ERK/MAPK expression in the SCN with aging.

## 6. AMPK Signaling

AMPK has an important role in energy metabolism and is activated in conditions of low energy [137,138]. It is a serine/threonine protein kinase formed by a catalytic α subunit and regulatory β and γ subunits. Each subunit has several isoforms, which are differently expressed in separate tissues and have diverse cellular localization [139]. Increased AMP/ADP ratio stimulates AMPK by allosteric regulation. AMPK activation is dependent on the energy status and the action of upstream stimulatory and inhibitory signaling pathways [140]. AMPK is activated by phosphorylation of upstream kinases including the tumor suppressor liver kinase B1 (LKB1), Ca^2+^/calmodulin-dependent protein kinase α, and β (CaMKKα and CaMKKβ), protein phosphatase-2Cα (PP2Cα) [139,140].

AMPK facilitates energy manufacture from glucose and fatty acids in stress conditions and suppresses synthesis of proteins, cholesterol, and glycogen [137,138,139,140]. Caloric restriction can elevate AMPK expression whereas food excess decreases AMPK activity and simultaneously induces insulin resistance and the emergence of metabolic syndrome, including such symptoms as obesity, diabetes, and cardiovascular diseases. AMPK activity decreases with aging, elevating the aging process [141,142].

AMPK indirectly inhibits the NF-κB pathway, activates FoxO signaling, and autophagy. AMPK can suppress the action of mTOR complex 1 (mTORC1) either by direct phosphorylating the Raptor, a regulatory component of mTORC1, or by the phosphorylation of tuberous sclerosis protein 2 (TSC2), which consequently inhibits the mTOR activity [141,142]. In turn, S6K1, a downstream target of the insulin/IGF-mTOR pathway, inhibits AMPK activity. S6K1 knockout activates AMPK signaling and increases the lifespan. AKT also inhibits AMPK signaling (Figure 3) [143,144].

In the hypothalamus, AMPK activation drives feeding by modulating mitochondrial fatty acid oxidation and intracellular levels of reactive oxygen species [145,146]. AMPK activity is inhibited in the ARN and PVN by leptin, and in multiple hypothalamic regions by insulin, high glucose, and refeeding [147,148]. Ghrelin administration induced a time-dependent AMPK increase in VMN [149]. There is an increase in hypothalamic AMPK activity in rats with diet-induced obesity, and the increased AMPK activity is associated with oxidative stress and inflammation in the hypothalamus [150]. The baseline pAMPK level was increased with age in the rat hypothalamus, whereas the total AMPK level remained unchanged. The pAMPK/AMPK ratio was also significantly higher in the aged rats [151].

Metformin, a well-known anti-aging drug, is also an activator of AMPK at the periphery, reducing glucose levels in type 2 diabetes. Other phytochemical geroprotectors such as berberine, quercetin, and resveratrol, have also been reported to activate AMPK signaling [141,142,152]. Recently, strong evidence was obtained that the important target for metformin is the hypothalamus, and its effects on the hypothalamic signaling plays a significant role to metformin-induced improvement of metabolic disturbances and increase in lifespan [153].

Metformin does not stimulate and even reduces AMPK activity in the hypothalamus, in contrast to peripheral tissues, which results in the activation of thermogenesis and a reduction in body weight and adiposity [154,155]. In the hypothalamus of obese agouti-mice, AMPK and STAT3 activities were increased, and metformin treatment stabilized the AMPK and STAT3 levels, augmented the activity of AKT, and increased POMC expression in the hypothalamus [61,155].

## 7. JAK/STAT Signaling

The JAK/STAT pathway mediates signals from many hormones and cytokines including GH, leptin, erythropoietin, and cytokines. JAK/STAT proteins are involved in the control of a variety of biological processes including proliferation, apoptosis, the immune response, inflammation, food intake, and energy homeostasis [156,157].

Upon the binding of a ligand to its cognate receptor, receptor-associated JAKs are dimerized and activated through autophosphorylation. Inactive cytosolic STAT monomers are transformed into active homodimers, heterodimers, or tetramers after being phosphorylated by JAKs. The active STATs can then translocate into the nucleus where they act as transcription factors to regulate gene expression. [156,157].

In mammals, there are four members of the JAK family: JAK1, JAK2, JAK3, and TYK2, and seven members of the STAT family: STAT1–4, STAT5A/B, and STAT6. Different combinations of JAKs and STATs are stimulated with a high specificity by various cytokines or growth factors. Other kinases, including Flt3R and pyruvate kinase, can phosphorylate STATs in addition to JAKs (Figure 3) [93,156,157].

JAK2, STAT3, and STAT5 are necessary for signaling through both the growth hormone (GH) and leptin receptors. STAT5 is the predominant STAT protein activated by GH [31,158], STAT3 and STAT5 mediate leptin-induced anorexic effects [158,159]. In addition to JAK2, activation of leptin receptors directly or indirectly triggers multiple signaling pathways including MAPK, FOXO1, AMPK, and others [159]. GH except JAK2 can activate Lyn (SRC family kinase member). Lyn stimulation by GH was shown to activate MAPKs. GH has also been shown to stimulate the PI3K pathway, probably through phosphorylation of the IRS [160]. Additionally, many impacts of the AKT/mTOR pathway on the immune system and aging are performed via STAT3 signaling [122].

In the hypothalamus, leptin elevates the transcription of suppressor of cytokine signaling 3 (SOCS3). SOCS3 suppresses leptin actions by a negative feedback loop [161]. In the ARN, STAT3 phosphorylation facilitates the leptin-mediated transcriptional regulation of POMC, AgRP, and NPY by binding to the promoters, which activates POMC and inhibits AGRP expression, thereby reducing food intake and elevating energy consumption [159]. In mice, neural-specific STAT3 knockout and mutation of leptin receptors that do not bind STAT3 leads to hyperphagia, obesity, reduced energy consumption, and augmented mRNA level of AGRP [155,160]. 

Exogenous leptin increases the satiating effect of GLP-1. The leptin receptors deficiency in neuronal cells expressing GLP-1 located in the solitary tract nucleus resulted in hyperphagia and obesity [26].

Basal levels of phosphorylated STAT and total STAT protein were not significantly different among young and aged rats [152,161]. However, leptin receptor expression was higher in the young compared with aged rats. These data indicate that aged rats demonstrated reduced signal transduction in response to centrally administered leptin [162].

## 8. Sirtuins

Sirtuins (SIRTs) are an evolutionarily conserved family of NAD^+^-dependent deacylases that play a key role in longevity in various organisms, including invertebrates and vertebrates [163]. The mammalian sirtuin family consists of seven members, SIRT1–7. SIRT2 is observed in the cytoplasm, while SIRT1, SIRT6, and SIRT7 are located in the nucleus [163,164]. 

SIRT isotypes vary in their functional activity: SIRT1, SIRT2, SIRT3, and SIRT-7 mainly display properties of (NAD^+^)-dependent deacetylase; SIRT4 and SIRT6 possess activities of deacetylases and ADP-ribosyltransferases. SIRT5, in addition to deacetylase activity, has functions of demalonylase and desuccincylase. SIRTs possess histone deacetylase activity, which is (NAD^+^)-dependent. Since NAD^+^ is a significant redox signaling molecule, SIRTs control antioxidant and redox signaling cellular pathways [164,165]. Due primarily to their impact on the p53 and FOXO activities, SIRTs play a significant role in cellular homeostasis and apoptosis [164,165]. SIRT1 suppresses the NF-ĸB signaling (Figure 3), increases the activity of the key antioxidant enzymes, and has a variety of other effects on glucose and lipid metabolism [163,164,166]. The interaction between neuroinflammation, neurodegeneration, and metabolic changes has been shown to be heavily dependent on SIRTs [167].

SIRT1 expression decreases with age in the human liver, heart, kidneys, brain, and lungs. Overexpression of *Sir2* in yeasts, or its homolog SIRT1 in animals, can increase lifespan [168,169]. The geroprotective properties of SIRT1, 2, 3, 6, and 7 are mediated by a variety of cellular signal cascades. The geroprotective effect of SIRT1 is regulated via p53, NF-κB, mTOR, PGC1α, and FOXO signal pathways [169,170,171]. Sirtuins are also required for caloric restriction-induced life span extension [171,172].

Resveratrol, a natural polyphenolic compound and potent geroprotector, allosterically modifies the activity of SIRT1. On the other hand, resveratrol also directly targets proteins other than SIRT1, such as AMPK and cAMP phosphodiesterases [169,171,173]. 

The level of SIRT1 decreases in the whole hypothalamus and separately in the ARN, VMN, DMN, and SCN with advanced age [171,174]. Supporting SIRT1 activity in the SCN could delay aging and extend lifespan [171,175]. In male and female transgenic mice overexpressing SIRT1 (BRASTO mice) in the DMN and LHA, the lifespan was increased [171,176]. Hypothalamic SIRT1 affects insulin sensitivity, systemic glucose, and lipid metabolism in the peripheral organs. SIRT1 in POMC neurons is required for normal adaptations against diet-induced obesity [177]. 

## 9. Nitric Oxide

Nitric oxide (NO) is a gaseous signal molecule participating in numerous brain functions including behavior, learning, memory, sleep, feeding, pain, and sexual activity [178,179]. NO is produced by NO synthase (NOS). There are three NOS isoforms: neuronal NOS (nNOS or NOS1), inducible NOS (iNOS or NOS2), and endothelial NOS (eNOS or NOS3). Ca^2+^ influx through NMDA receptors is necessary for nNOS activation and the subsequent generation of NO. Once NO is produced, it can easily diffuse through membranes and bind to the NO receptor, soluble guanylate cyclase, which synthetizes the second messenger cGMP [178,179,180].

In the hypothalamus, nNOS expression is largely restricted to areas involved in the control of bodily functions, including metabolism and reproduction. nNOS-expressing neurons are located in the preoptic region, supraoptic, ventral premamillary nucleus, DMN, VMN, PVN, and LHA [181,182,183].

Aging is associated with high levels of reactive oxygen species and the NO metabolite peroxynitrite in the brain [184,185]. Redundant NO production causes cell death, but augmented NO release can prevent apoptosis [186]. nNOS expression and activity increases in neurodegenerative diseases, epilepsy, inflammation, and ischemia [187,188]. There is some evidence that increased NO production in the hippocampus reduces hippocampal neurogenesis [186]. While nNOS-expressing neurons are thought to be more resistant to neurotoxic effects, NO can serve a protective role throughout aging [184].

In young rats, weakly nNOS-immunopositive neurons were found in the DMN and VMN, but were missing in the ARN. In aged rats, the number of neurons containing nNOS significantly increased in the DMN and VMN, and a large number of nNOS-immunoreactive neurons was found in the ARN [183].

## 10. Hypothalamic Inflammation in Aging

In the aged brain, augmented levels of pro-inflammatory cytokines including TNF, interleukin (IL) 1β, IL 6, and reduced levels of anti-inflammatory cytokines, such as IL 2 and IL 10 are observed. Activation of the signaling molecules such as Toll-like receptor 4 (TLR4), myeloid differentiation primary response gene 88 (MyD88), nuclear factor kappa B (NF-κB), inhibitor of nuclear factor kappa B kinase subunit beta (IKK), and c-Jun N-terminal kinase (JNK) is associated with the development of the obesity and the metabolic syndrome in the mediobasal hypothalamus [5,8,189]. The NF-κB signaling acts in close co-operation with a number of pathways, including JAK-STAT3 and insulin/IGF-1 [125]. The NF-κB system is stimulated by the PI3K/AKT/IKKα/β pathway. Nevertheless, it is known that InsR/IGF-1R can also affect the NF-κB complex through the RAF-1 and MAPK pathways [189].

Latest studies have also revealed a crucial role of this hypothalamic microinflammation in the aging processes. In the mice mediobasal hypothalamus, suppression of the NF-κB signaling extended the lifespan, whereas stimulation of the NF-κB pathway reduced the duration of life [5,190]. GABA-ergic and BDNF-expressing neurons of the mediobasal hypothalamus are vulnerable to hypothalamic inflammation induced by a high-fat diet. In this case, hypothalamic astrocytes miss their terminal processes, and reduce GABA uptake. Elevated GABA concentration in the intercellular space suppressed BDNF-expressing neurons [191,192]. Vice versa, the injection of BDNF viral vector into the mediobasal hypothalamus of middle-aged mice reduced the expression of inflammatory genes in the hypothalamus and prevented age-associated metabolic disturbances [193]. In the ARN, stimulation of GABA_B_ receptor in POMC neurons prevented hypothalamic inflammation, obesity, and insulin resistance in mice [194]. Continued hypothalamic inflammation activates glutamatergic transmission, which can lead to hypertension as a part of metabolic syndrome [195].

GLP-1 also exerts its anti-inflammatory response by expressing GLP-1R in astrocytes and glial cells during neuroinflammation. This anti-inflammation effect of GLP-1 is realized via IGF-1 receptor and NF-κB signaling [71].

In rodents, NF-κB signaling suppresses GnRH gene transcription that can induce the age-associated GH decrease. However, intracerebroventricular or subcutaneous GnRH treatment prevented age-related decrease in hypothalamic hippocampal neurogenesis, restored muscular strength, skin thickness, bone mass, and collagen integrity in the caudal tendon in mice [8].

## 11. Conclusions Remarks

Based on Dillman’s elevation theory of aging, senescence is associated with an increase in the threshold of hypothalamic responsiveness to homeostatic signals. The aging process in the hypothalamus is accompanied by impaired intracellular signaling including PI3K/AKT/mTOR, ERK/MAPK, AMPK, Sirtuins, NF-κB, NO, and disturbances in the release of neurotransmitters (e.g., GnRH, NO, NPY, α-MSH). There are some differences between disturbances of the signaling pathways in the hypothalamus and periphery. The activity of ERK/MAPK, NF-κB, and nNOS increases, SIRT1 decreases, and the expression of AKT and STAT3 does not change in the aging hypothalamus in rodents (for a short summary, see Table 1). In addition, there are some differences in signaling in functionally antagonistic groups of hypothalamic neurons, for example POMC and AGRP neurons. The mechanisms of hypothalamic aging are still not fully understood, despite recent advances in our understanding of the organization of hypothalamic networks. Utilizing a variety of research techniques, such as molecular biology and genetics, will aid in the explanation of this issue.

## Figures and Tables

**Table 1:** The summary of changes in signaling pathways during aging.

Signaling Pathway	Aging Tissues	Aging Hypothalamus
AKT/mTOR	Differential actions in separate organs	No changes
STAT3	No changes	No changes
ERK/MAPK	Decreased	Increased
AMPK	Decreased	Increased
Sirtuins	Decreased	Decreased
NF-κB	Increased	Increased
NO	Increased	Increased

## Data Availability

Not applicable.
